# Innovative approaches in stem cell therapy: revolutionizing cancer treatment and advancing neurobiology – a comprehensive review

**DOI:** 10.1097/JS9.0000000000002111

**Published:** 2024-10-08

**Authors:** Dhrupad Banerjee, Arghya Bhattacharya, Abhijeet Puri, Shubham Munde, Nobendu Mukerjee, Popat Mohite, Syeda W. Kazmi, Abhishek Sharma, Taha Alqahtani, Humood Al Shmrany

**Affiliations:** a Department of Microbiology, Ramakrishna Mission Vivekananda Centenary College, Rahara, Khardaha, West Bengal, India; b Department of Pharmacology, Bengal School of Technology (a college of pharmacy), Sugandha, West Bengal, India; c AETs St. John Institute of Pharmacy and Research, Palghar, Maharashtra, India; d Center for Global Health Research, Saveetha Medical College and Hospital, Saveetha Institute of Medical and Technical Sciences, Chennai, India; e Chandigarh Pharmacy College, Chandigarh Group of Colleges, Jhanjeri, Mohali, Punjab, India; f Department of Medicine, National Institute of Medical Sciences, NIMS University Rajasthan, Jaipur, India; g Department of Pharmacology, College of Pharmacy, King Khalid University, Abha, Saudi Arabia; h Department of Medical Laboratory Sciences, College of Applied Medical Sciences, Prince Sattam bin Abdulaziz University, Alkharj, Saudi Arabia

**Keywords:** autoimmune diseases, cancer, exosomes, mesenchymal stem cells, nanotechnology, neurobiology

## Abstract

Stem cell therapy represents a transformative frontier in medical science, offering promising avenues for revolutionizing cancer treatment and advancing our understanding of neurobiology. This review explores innovative approaches in stem cell therapy that have the potential to reshape clinical practices and therapeutic outcomes in cancer and neurodegenerative diseases. In this dynamic and intriguing realm of cancer research, recent years witnessed a surge in attention toward understanding the intricate role of mesenchymal stem cells (MSCs). These cells, capable of either suppressing or promoting tumors across diverse experimental models, have been a focal point in the exploration of exosome-based therapies. Exosomes released by MSCs have played a pivotal role, in unraveling the nuances of paracrine signaling and its profound impact on cancer development. Recent studies have revealed the complex nature of MSC-derived exosomes, showcasing both protumor and antitumor effects. Despite their multifaceted involvement in tumor growth, these exosomes show significant promise in influencing both tumor development and chemosensitivity, acting as a pivotal factor that increases stem cells’ potential for medicinal use. Endogenous MSCs, primarily originating from the bone marrow, exhibited a unique migratory response to damaged tissue sites. The genetic modification of stem cells, including MSCs, opened avenues for the precise delivery of therapeutic payloads in the milieu around the tumor (TME). Stem cell therapy offers groundbreaking potential for treating neurodegenerative and autoimmune disorders by regenerating damaged tissues and modulating immune responses. This approach aims to restore lost function and promote healing through targeted cellular interventions. In this review, we explored the molecular complexities of cancer and the potential for breakthroughs in personalized and targeted therapies. This analysis offers hope for transformative advancements in both cancer treatment and neurodegenerative disorders, highlighting the promise of precision medicine in addressing these challenging conditions.

## Introduction

Highlights

- Stem cell therapies are advancing with nanotechnology, enabling targeted delivery systems that enhance precision in attacking cancer cells while sparing healthy tissue, potentially transforming cancer treatment outcomes.
- Stem cells are showing promise in neurobiology by offering avenues for replacing damaged neurons and promoting neural regeneration, which could lead to groundbreaking treatments for neurodegenerative diseases like Alzheimer’s and Parkinson’s.
- The integration of nanotechnology in stem cell research is facilitating innovative approaches such as nanocarriers for drug delivery and molecular imaging, enhancing therapeutic efficacy and monitoring capabilities in both cancer treatment and neurobiology.

The investigation of exosome-based therapeutic approaches derived from MSCs in the context of cancer is currently a topic of active discussion and exploration. This emerging field has gained significant attention in recent years, shedding light on the complex role that MSCs play in either suppressing or promoting tumors in various experimental models. The use of exosomes released by MSCs has become an intriguing avenue for understanding paracrine signaling and its profound influence on different aspects of cancer development. A study by Vallabhaneni *et al*. reveals that exosomes from bone marrow-derived mesenchymal stem cells (BMMSCs) of multiple myeloma patients contribute to the enhancement of multiple myeloma tumor growth^1^. The influence of exosomes derived from bone marrow mesenchymal stem cells (BMMSCs) extends to the growth of gastric or colon tumors by stimulating the production of vascular endothelial growth factor (VEGF) in tumor cells. These exosomes also play a crucial role in promoting advancement and migration in nasopharyngeal carcinoma by triggering the epithelial–mesenchymal transition and activating the FGF19-FGFR4-dependent ERK signaling cascade. Notably, a study by Bruno *et al*. found that exosomes from human BMMSCs exhibit inhibitory effects on the growth and survival of multiple human tumor cell lines. Furthermore, several types of cancer cells undergo apoptosis when exposed to exosomes produced by MSCs overexpressing the TRAIL gene, offering a promising avenue for therapeutic intervention^2^.

The intriguing dual nature of MSC-derived exosomes persists, as certain subsets demonstrate antitumor effects. According to a study by Bruno *et al*., human BM-MSC exosomes not only prevent different human tumor cell lines from growing and surviving, but they also have comparable effects in NOD/SCID mice models. Furthermore, several cancer cell types undergo apoptosis when exposed to exosomes produced by MSCs overexpressing the TRAIL gene, providing a potential therapeutic avenue. Extending beyond their impact on tumor growth, MSC-derived exosomes significantly influence tumor chemosensitivity^3^. Research by Lou *et al*. suggests that exosomes derived from human umbilical cord MSCs cause gastric cancer cells to become resistant to 5-fluorouracil in a subcutaneous xenograft tumor model in BALB/c nu/nu mice. This resistance is achieved through the antagonization of apoptosis and the enhancement of the expression of proteins linked to multidrug resistance. In a different context, Lou *et al*.’s study illustrates that exosomes from BMMSCs transfected with anti-miR-9 effectively reverse chemoresistance in temozolomide-resistant glioblastoma multiforme cells^4^.

**Figure 1:**
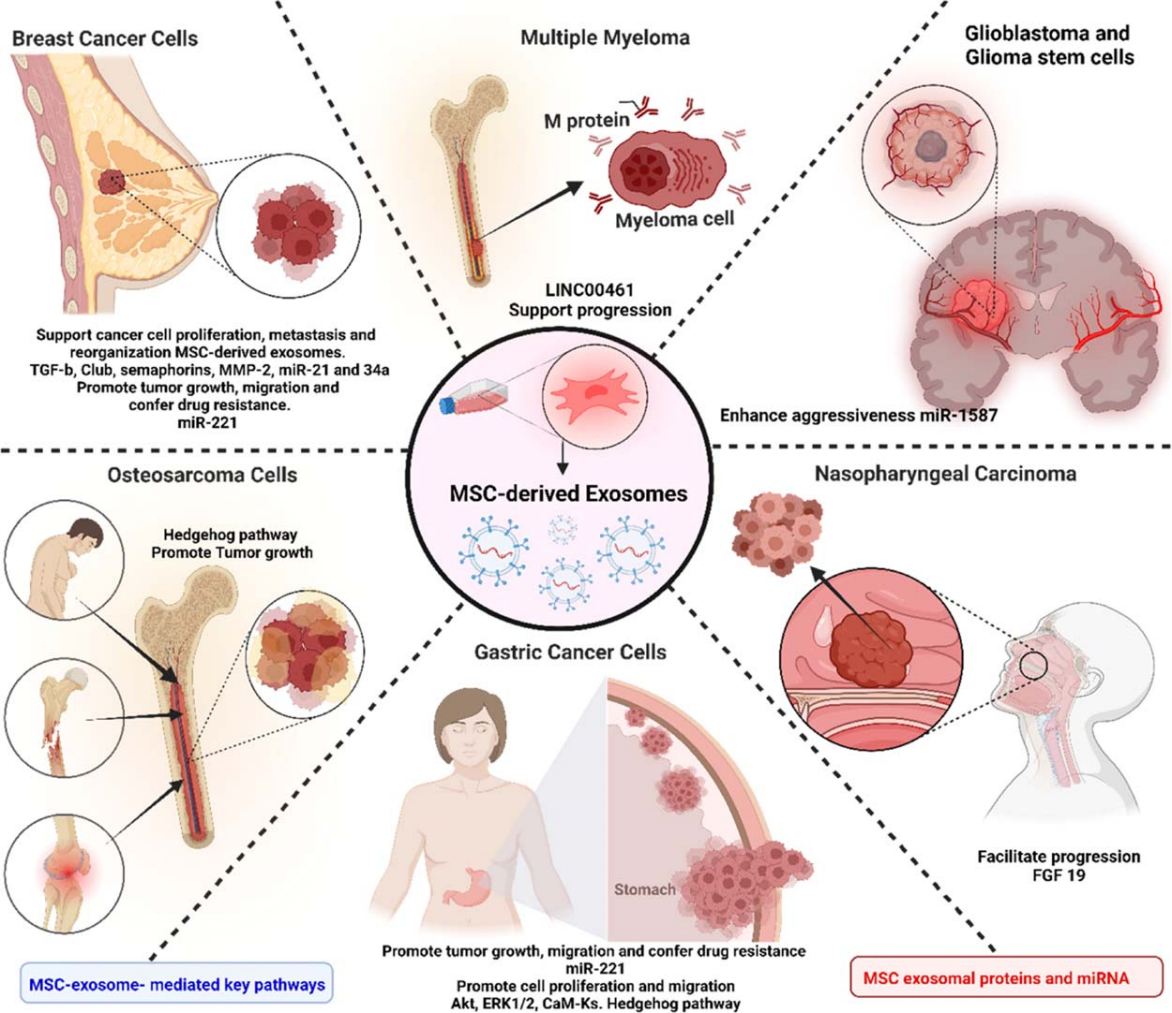
MSC exosomes can significantly enhance tumor growth and migratory capacity across a broad spectrum of cancers, including gastric, breast, and osteosarcoma. Mechanistically, it is mediated by the transfer of microRNAs (e.g. miR-221) and proteins (e.g. TGF-β, MMP-2) from MSC exosomes to cancer cells. These transferred molecules activate oncogenic signaling pathways (e.g. Akt, ERK1/2, Hedgehog) within the recipient cancer cells. MSC exosomes also contribute to the development of drug resistance and disease progression in malignancies like multiple myeloma and glioblastoma. (Adapted from Zhao *et al*.^2^ under CC BY 4.0, Hindawi Publisher).

Despite the intricate MSC-derived exosomes’ dual function, their substantial promise in influencing both tumor development and chemosensitivity is evident. However, the potential application of these exosomes in cancer therapy requires careful consideration due to the complex and multifaceted nature of their involvement in tumor growth. MSC exosomes can significantly enhance tumor growth and migratory capacity across a broad spectrum of cancers, including gastric, breast, and osteosarcoma (Fig. 1). Simultaneously, the demonstrated role of these exosomes and their potential in tumor suppression makes them attractive options for cancer therapy without the use of cells. The goal of this review is to offer an extensive understanding of the mechanisms governing MSC-derived exosomes, offering valuable insights into their intricate roles in the development of cancer and their potential as medicinal substances^2^.

## Stem cell therapies in cancer treatment

Cancer poses a significant global health challenge, impacting millions annually and ranking as the second most common cause of death in various regions, closely trailing cardiovascular diseases. Despite considerable progress in managing cardiovascular diseases, projections suggest that it will soon be surpassed by cancer as the leading cause of death. This is particularly evident with the aging population contributing to the escalating burden of cancer-related cases and deaths^5^. In 2002, the GLOBOCAN database reported an alarming 10 862 496 new cancer cases worldwide. Gender distribution revealed a disparity, with 53.4% occurring in males and 46.6% in females. Geographically, Asia accounted for nearly 45% of cases, Africa (6%), Latin America (7%), North America (15%), and Europe (26%), in that order. Globally, the most common cancer sites included the lung, colon, and stomach, each presenting unique challenges for diagnosis and treatment^6^.

**Figure 2:**
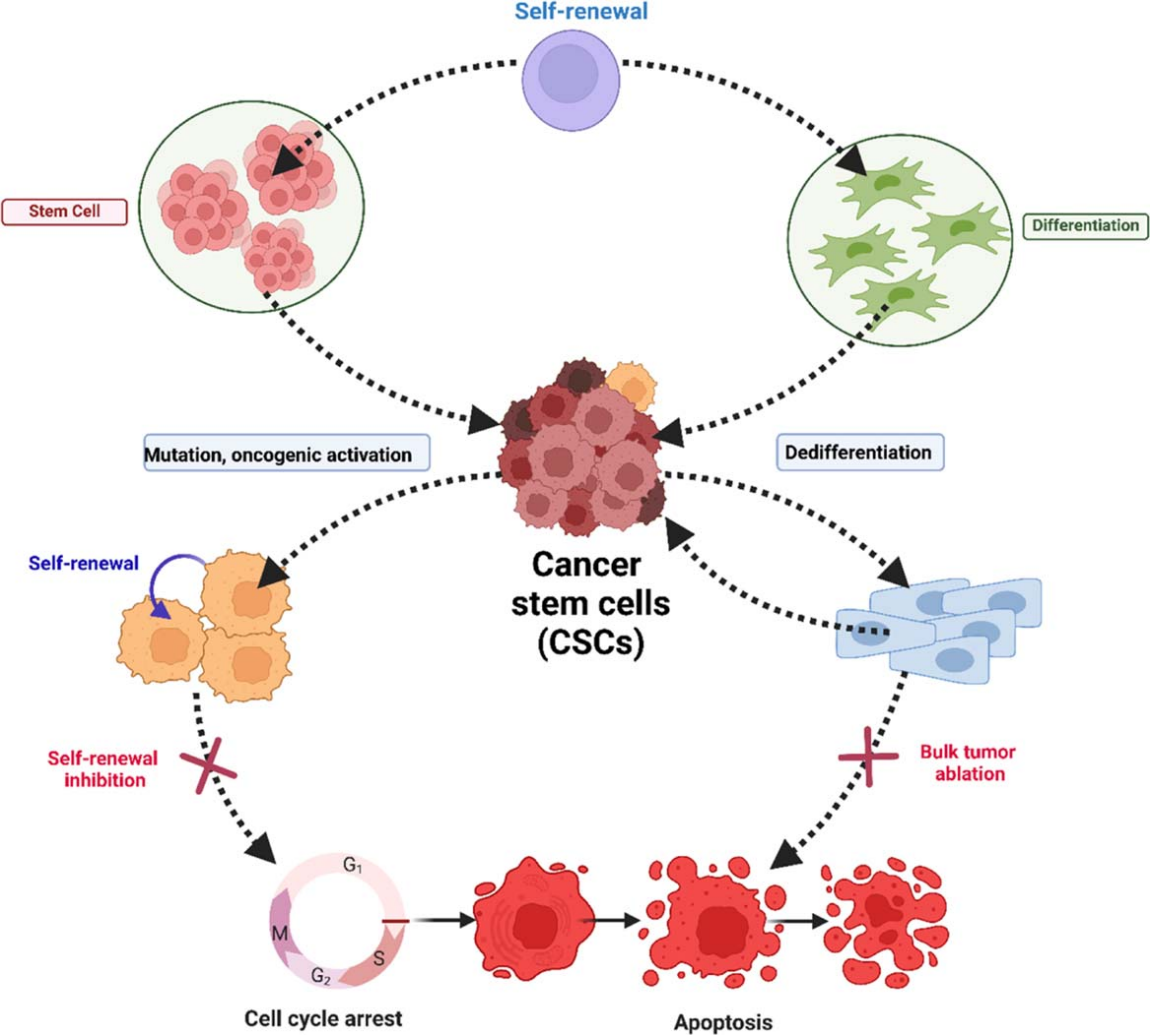
The origin of CSCs and combinational therapy of CSC targeting and bulk tumor ablation. (Adapted from Phi *et al*.^7^ under CC BY 4.0 from Hindawi Publisher).

The complexity of cancer research has heightened with the emergence of cancer stem cells (CSCs), tracing back to stem cells and sharing characteristics with normal stem cells. Their ability to proliferate in microenvironments underscores their pivotal role in sustaining cancer growth, emphasizing the importance of targeting them for effective cancer elimination (Fig. 2). A specific subset of CSCs is known for overexpressing. There has been evidence of CD-47 on the cell surface of lung, liver, and pancreatic cancers. CD-47 acts as an immunosuppressive signal, inhibiting attacks from macrophages and serving as an immune checkpoint blockade. Strategies targeting CD-47, often combined with PD-L1, have shown promise in enhancing immunotherapy against circulating tumor cells, resulting in a greater decrease of solid tumors in experimental mouse models^8^.

To address challenges posed by CD-47 overexpression, various strategies based on pharmacology and nanomedicine have been developed. Antibodies like Hu5F9-G4 and rituximab target CD-47, enabling macrophages to effectively eliminate cancerous cells. Clinical studies on patients with non-Hodgkin lymphoma treated with these antibodies have shown significant symptom relief, with more than 60% of patients showing signs of a full or partial recovery^9^. Targeting CD-47 on cancer stem cells is significant since it has displayed no detectable side effects in humans, presenting a promising avenue for immunotherapy against various forms of cancer^9^.

The identification of CSC-surface molecules offers significant potential as targets for treatments involving cytotoxicity, especially those using neutralizing antibodies. Combined therapies have been explored to prevent tumor recurrence and suppress the CSC population. In drug-resistant triple-negative breast cancer (TNBC) cells, the TGF-β pathway of CSCs has been targeted. A combined therapy involving a SMAD-4 siRNA, a TGF-β type II receptor neutralizing antibody, and a TGF-β type 1 receptor kinase inhibitor, and paclitaxel chemotherapy has shown promise. This approach blocked the recruitment of IL-8, inhibiting the growth of populations of chemotherapy-resistant CSCs^7^.

Additionally, a hallmark for cancer stem cells has been found in aldehyde dehydrogenase 1 (ALDH1), offering another potential target for molecular therapy. Thus, the global burden of cancer necessitates innovative approaches to its treatment. Targeting CSCs, particularly through the CD-47 pathway, has demonstrated encouraging preclinical and clinical studies. Combined therapies addressing various pathways associated with CSCs offer a comprehensive strategy to inhibit tumor recurrence and drug resistance. Further exploration of molecular markers like ALDH1 provides additional avenues for advancing cancer treatment modalities^10^.

Shifting the focus to the role of genetic modification in oncology, it plays a pivotal role in augmenting the medicinal possibilities of stem cells. Endogenous mesenchymal stem cells (MSCs), predominantly originating from the bone marrow, exhibit a distinctive migratory response toward damaged tissue sites^11^. A complicated interaction between chemokines and signaling pathways, including the expression of many chemokine receptors including CCR1, CCR2, CXCR4, and others, is responsible for this tropism. Furthermore, MSCs have a variety of cell adhesion molecules that enable them to engraft into certain target tissues, which is an essential component of efficient mobilization. MSCs migrate out of the injection site and into the tumor microenvironment (TME) after transplantation, where they engraft to a variety of target cells^12^. The genetic engineering of multipotent stem cells, or MSCs, opens avenues for the precise delivery of therapeutic payloads within the TME.

Prodrug-converting enzymes, such as those used in gene-directed enzyme prodrug treatment (GDEPT), growth factors, chemotactic cytokines, interleukins, interferons, and virally transduced MSCs and neural stem cells (NSCs) have all been demonstrated to produce these enzymes. With the use of non-endogenous enzymes made by genetically altered stem cells, GDEPT makes it possible to transform prodrugs that are not poisonous into their active forms. Gliomas, medulloblastomas, and other brain cancers may benefit from this treatment strategy, which takes advantage of MSCs’ transient manipulation of tight junctions inside the blood-brain barrier (BBB)^13^.

**Figure 3:**
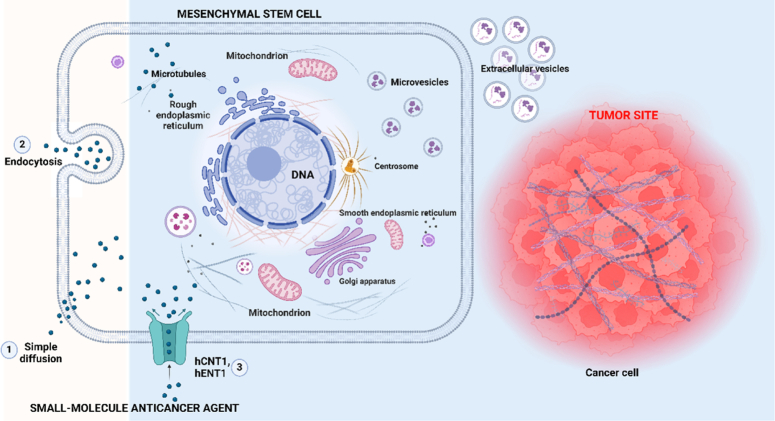
Schematic representation of the delivery of therapeutic agents by MSC to tumor site via various mechanisms: 1. Simple diffusion, 2. Endocytosis, 3. Transporters such as hCNT1, hENT1. Small-molecule anticancer agents are enclosed in vesicles produced by MSCs. The existence of EVs between MSCs and cancer cells implies that a vesicular system can transport the small-molecule anticancer agent to the cancer cells. (Adapted from Szewc *et al*.^14^ and Jia *et al*.^15^).

Given the inherent qualities that make them tumor tropism, MSCs are excellent therapeutic agents. Many biomarkers are provided by intrinsic tumor tropism, which can be used as targets for nanoparticles, allowing for in-vivo imaging through clinically relevant modalities like nuclear imaging and magnetic resonance imaging (MRI). The manipulation of tight junctions within the BBB by MSCs facilitates their seamless traversal into the cortex, employing ways for tumor tropism to enter and destroy brain tumor cells. This dual functionality positions MSCs as versatile vehicles for targeted therapy and diagnostic imaging in the complex landscape of oncology^14^ (Fig. 3).

**Figure 4:**
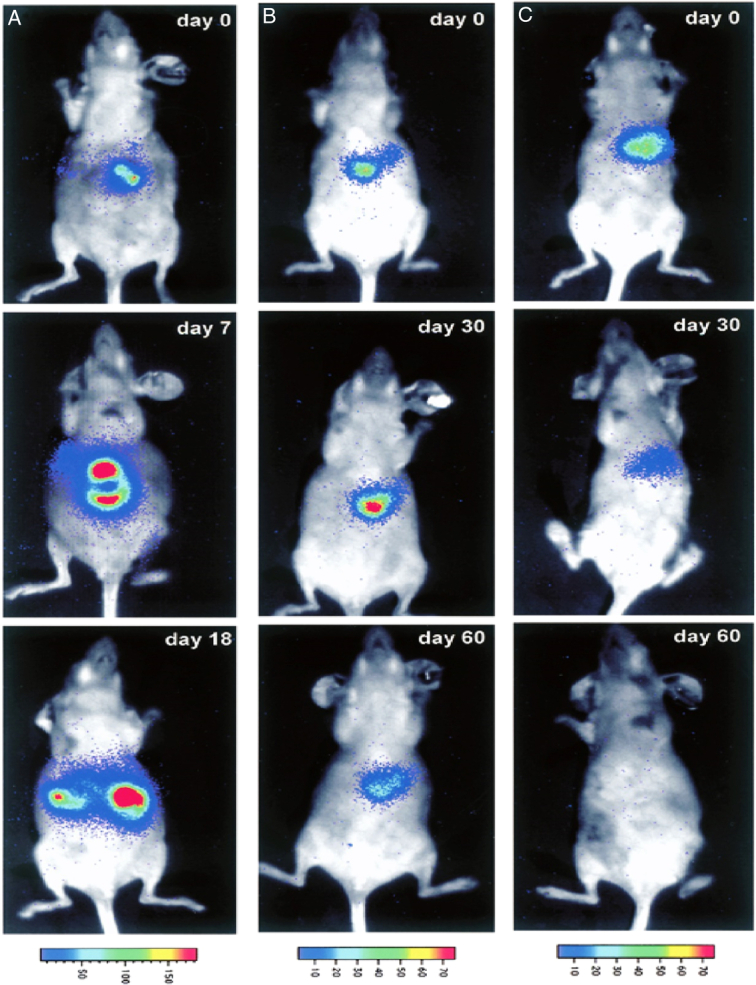
The impact of 5-FC on the bioluminescence of yCD/HT-29 luciferase expressing intrahepatic tumors. Tumors were found in mice with 0% content. (A) a 10% rate, (B) or entirely. The tumor burden of yCD/HT-29luc cells was evaluated by bioluminescence after treatment with 5-FC, following the procedure outlined in the ‘Materials and methods’ section. A range of detected photons is responsible for the colors in the rainbow spectrum. (Adapted from Nyati *et al*.^17^).

In a groundbreaking comparative study, MSCs were employed as a medium to assess the effects of minute modifications to several enzyme/prodrug systems, such as nitroreductase/CB1954 (NTR/CB1954), yeast cytosine deaminase/5-fluorocytosine (yCD/5-FC), and thymidine kinase/ganciclovir (TK/GCV), on therapeutic outcomes. Genetically modified four suicide genes, including TK, yeast cytosine deaminase, uracil phosphoribosyl transferase (yCD:UPRT), and nitroreductase (NTR), were expressed consistently by MSCs^16^. Using SKOV3 cell models to evaluate the anticancer efficacies *in vivo*, yCD:UPRT/5-FC was shown to be the most efficient enzyme/prodrug system among those tested, demonstrating the potential of this theranostics imaging platform. The global burden of cancer necessitates constant innovation, and these avenues of research present promising strides toward more effective and comprehensive cancer treatment modalities. As we delve deeper into the molecular and genetic intricacies of cancer, the potential for breakthroughs in personalized and targeted therapies continues to grow, offering hope in the battle against this pervasive and complex disease^17^. The impact of 5-FC on the bioluminescence of yCD/HT-29 luciferase expressing intrahepatic tumors is presented in Figure 4.

## CRISPR technology in cancer therapy

CRISPR (Clustered Regularly Interspaced Short Palindromic Repeats) technology has advanced, precise DNA sequence adjustments are now possible, making it a breakthrough tool for genome editing^18^. A guide RNA is used by the CRISPR–Cas9 system, which originated from an immunological defense mechanism seen in bacteria, to direct the Cas9 endonuclease to certain genomic locations where it makes a double-stranded break^19^. After that, these breaks are fixed either non-homologous end joining or homology-directed repair, which produces targeted insertions or substitutions or gene knockouts^20^. Since CRISPR technology allows for quick and effective genetic modification, its use in a wide variety of animals and cell types has transformed scientific study^21^. Modifying the genome with CRISPR has great potential to advance cancer research and provide new treatment strategies^22^.

Tumor suppressors, stability genes, and oncogenes are among the genes that frequently accumulate mutations that disrupt normal cellular activity and cause cancer^23^. Replicating these mutations is necessary for modeling cancer to examine the effects on treatment sensitivity, metastasis, and carcinogenesis^24^. The homologous recombination techniques used in conventional procedures are difficult and take a long time. Conversely, though, CRISPR uses readily created guide RNAs to quickly and precisely introduce deletions, chromosomal rearrangements, and knockouts^25,26^. This rapid development of animal models and isogenic cell lines offers previously unheard-of insights into the biology of cancer^27^. Moreover, CRISPR screens methodically identify genes necessary for cancer cell viability as well as possible therapeutic targets^28,29^.

**Figure 5:**
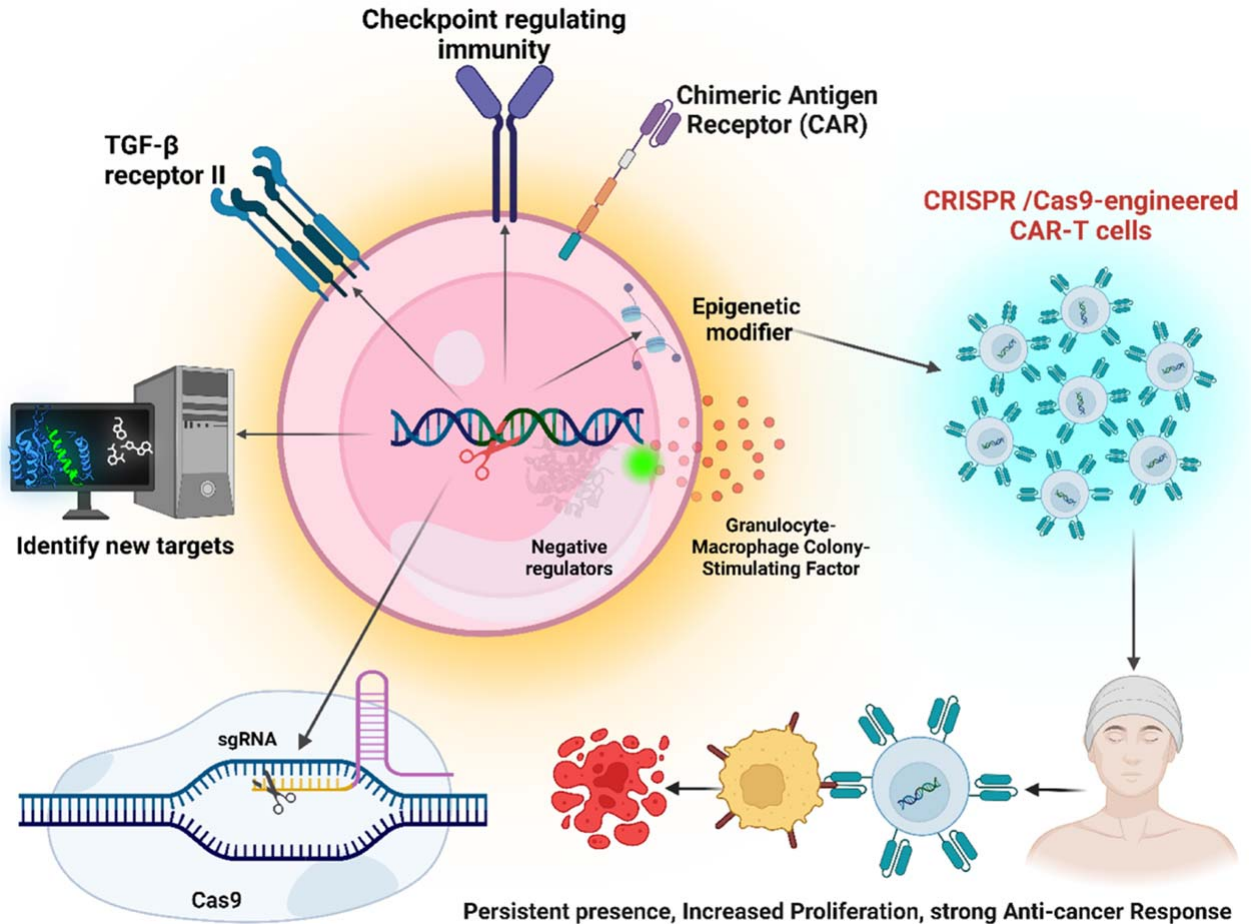
This schematic depicts the application of CRISPR–Cas9 gene editing to enhance the long-term persistence of CAR T cells in cancer immunotherapy. Potential strategies include (1) Disruption of T cell exhaustion-related genes (e.g. PD-1) to prevent CAR T cell inactivation. (2) Knock-in of genes promoting memory T cell formation (e.g. TCF1) for sustained antitumor response. (3) Modification of metabolic pathways to improve CAR T cell fitness and persistence within the tumor microenvironment. Overall, CRISPR–Cas9 offers a versatile toolkit to engineer next-generation CAR T cells with enhanced functionality and longevity for effective cancer therapy. (Adapted from Doudna and Charpentier^18^).

Beyond simulation, CRISPR enables the remedial editing of mutations that cause cancer^30^. Certain B cell malignancies can go into remission with CD19-targeted CAR-T therapy, and CRISPR can increase both the treatment’s safety and effectiveness^31^. Enhancing T cell editing to eliminate immunological checkpoints or fix functional flaws enhances responses against solid malignancies as well^32^. Natural killer cell engineering can be done precisely using CRISPR to create allogeneic immunotherapies^33^. Modifying hematopoietic stem cells can impart cancer resistance, which might stop relapses^34^. Base editors can fix oncogenic point mutations in nucleotide substitutions without causing double-strand breaks^35^. CRISPR thus has great promise for creating cancer-curative genomes and cell treatments. On the other hand, effective in-vivo delivery presents difficulties for clinical translation^19^. Additionally, ethical considerations call for close monitoring of appropriate usage^18^. However, CRISPR technology offers the potential to revolutionize drug discovery, cancer models, and treatment paradigms by facilitating quick and accurate genome modification. To fully exploit these benefits for patients, further developments are being made. The application of CRISPR–Cas9 gene editing to enhance the long-term persistence of CAR T cells in cancer immunotherapy (Fig. 5).

### CRISPR–Cas9

Because CRISPR–Cas9 is so precise, easy to use, and adaptable, it has quickly become the most sophisticated targeted genome editing technique^36^. The Cas9 endonuclease and single-guide RNA (sgRNA) comprise the CRISPR–Cas9 system. By modifying its complementary sequence, the 20-nucleotide sgRNA’s sequence may be engineered to target any chromosomal location^37^. Upon binding to its designated DNA target, the sgRNA mobilizes Cas9 to initiate a double-strand break at that location. After this, these breaks are fixed by native processes such as non-homologous end joining or homology-directed repair, which produce precise edits or gene knockouts, respectively^38^.

Since sgRNA-DNA complementarity determines the targeting specificity of CRISPR, it is easy to precisely change almost any sequence^39^. CRISPR may induce exact nucleotide changes, deletions, or insertions by supplying repair templates. Additionally, it permits effective multiplexing through the simultaneous use of numerous sgRNAs^40^. By fusing a catalytically dead Cas9 to transcriptional repressors or activators, CRISPR may control genes without changing the DNA sequence^41^. Precision editing is more versatile now that it can convert individual nucleotides programmable in addition to cleaving DNA using modified Cas enzymes like prime editors and base editors^42^.

CRISPR offers more simplicity and efficiency in design and delivery as compared to other technologies such as TALENs and zinc finger nucleases. Nonetheless, CRISPR can overlook certain base mismatches between the target and sgRNA, which might lead to off-target editing^43^. Novel sgRNA scaffolds, enhanced delivery strategies, and advanced Cas variants like SpCas9-HF1 all aid in lowering off-target effects and enhancing specificity^23^.

**Figure 6:**
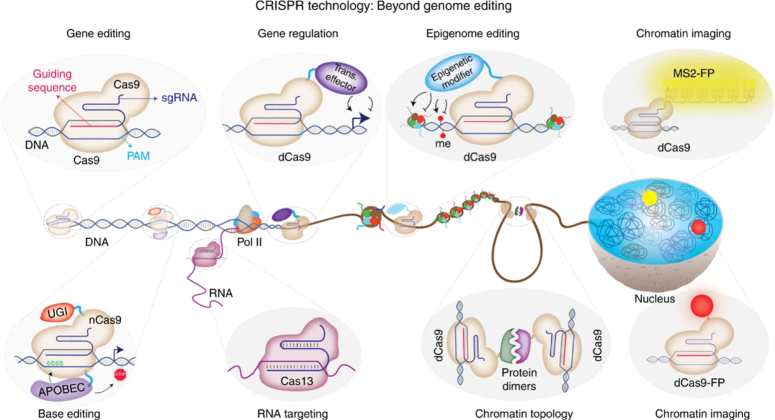
CRISPR–Cas expands beyond gene editing: modified Cas9 enzymes regulate genes (dCas9), edit chemical tags on DNA (epigenome editing), and visualize DNA in living cells. Engineered Cas9 nickases enable precise base editing without DNA breaks. New RNA-targeting CRISPR systems offer exciting possibilities for RNA manipulation. This versatile toolkit unlocks a new era of biological discovery. (Adapted from Adli^20^ under CC BY 4.0, Nature Publishers).

CRISPR has advanced genetic engineering due to its ease of use, low cost, and multiplexing potential^44^. CRISPR provides fast, accurate editing to analyze gene function, fix disease mutations, and enhance agricultural yields. Prolonged advancements in genome editing instruments hold out hope for broadening the scope of biological studies and facilitating groundbreaking medical interventions^45^ (Fig. 6).

### Development of precise gene editing tools like CRISPR–Cas9

The development of precise gene editing tools like CRISPR–Cas9 has made it possible to treat cancer in novel ways by modifying the genome. Mutations in important genes that regulate cell growth and survival are the root cause of cancer^24^. Curative medicines might be developed by fixing such mutations or changing genes to boost antitumor immunity^46^.

CRISPR-mediated oncogene knockdown reduces the viability and tumorigenicity of cancer cells *in vivo*, exposing new targets for therapeutic intervention^47^. Cancer cell lines undergo apoptosis when mutations affecting tumor suppressors like p53 are corrected^48^. CRISPR screens are useful for methodically identifying genes necessary for the growth of cancer cells in addition to medication combinations that work well together^49^.

Additionally, CRISPR permits immune cell modification to combat cancer. In preclinical models, PD-1 deletion improves T cell activation and solid tumor suppression^50^. Tumor immunity is enhanced by correcting mutations that lead to T cell malfunction, such as those in the IL2 receptor^51^. The cytotoxicity of modified CAR T cells is enhanced against solid tumors and leukemia in mice^52^.

Using CRISPR, it is possible to quickly insert engineered chimeric antigen receptors and T cell receptors to redirect Tcells against tumor antigens. Hematopoietic stem cells with modifications can provide resistance to malignancy. Improved antitumor activity is also shown by CRISPR-modified natural killer cells that have had their inhibitory receptors deleted. Base editors allow programmed single-base modifications without double-strand breaks for nucleotide substitutions, which permits the correction of oncogenic point mutations. For accurate insertions, deletions, and all transition alterations, prime editors provide even more freedom^49^. Nevertheless, bringing in-vivo gene editing to the clinic will not be easy due to effective delivery. Vigilant supervision is also necessary due to ethical concerns over human germline modification. However, CRISPR-based genetic medicines provide a groundbreaking opportunity for the management of cancer^34^.

### The advent of CRISPR gene editing technology

Given that the use of CRISPR gene editing technology can modify human genomes, several ethical questions have been raised by this development^53^. Important factors for the safe and appropriate application of CRISPR include:

- Germline editing: Modifying human gametes or embryos can result in genetic alterations that are inherited by subsequent generations^54^. Although there are still unidentified hazards and ethical concerns, this may one day be used to cure hereditary disorders^55^. Human germline modification for therapeutic purposes is optional at this point until the full ramifications are known.
- Safety: It is crucial to ensure patient safety, particularly in first-in-human studies^56^. Before entering the clinic, possible risks including immunogenicity, toxicity during delivery, and off-target effects need to be thoroughly investigated. Safety margins can be increased by optimizing dosage properly and restricting changes to genes that are not necessary^57^.
- Access and equity: Regardless of socioeconomic background, clinical uses of CRISPR should be inexpensive and available to all patient groups that require them^58^. Currently, widespread usage of treatments like CAR T cells is limited by patents and exorbitant costs. It is also critical to educate and raise public knowledge of the advancements in genome editing^59,60^.
- Enhancement: Changing human characteristics unrelated to treating a disease presents ethical questions about potential abuse for eugenics or enhancement^61,62^. Rules are required to avoid ‘designer babies’ and limit the application of CRISPR to genetic modification that is medically necessary^60^.
- Informed consent: To give appropriate informed permission, patients taking part in CRISPR studies need to be well informed about the possible risks, advantages, and limits^63^. Tracking long-term impacts also requires patient follow-up monitoring.

Scientists have urged for an ethical framework that involves worldwide collaboration, transparent research, and including specialists outside scientists, such as ethicists, patients, and legislators, to safely transfer CRISPR-based medicines^64^. CRISPR gene editing has revolutionary potential to address numerous genetic illnesses with responsible development.

## Recent advances in stem cell therapies

Recent advances have enabled more precise, personalized therapies against cancer, transforming the treatment landscape. Here we highlight emerging modalities including engineered cell therapies, targeted small molecules, combination strategies, and novel radiotherapy approaches.

### Adoptive cell therapies

The goal of adoptive cell transfer (ACT) is to use genetically modified or endogenous anticancer lymphocytes. Tumor-infiltrating lymphocytes (TILs) have been successfully expanded and isolated in melanoma patients, with response rates reaching 50% in recent studies^63,65^. Tumor specificity without genetic alteration is provided by TILs, which identify patient-specific neoantigens^50^. Cytokines, checkpoint inhibitors, and the introduction of tumor-specific TCRs or CARs are methods that may improve TIL treatment^66,67^.

While CAR T cell treatment can cause long-lasting remissions in some B cell malignancies, it has difficulties when treating solid tumors^68,69^. Solid tumors do not have limited antigens like the B cell antigen that CAR T cells target anti-CD19, which reduces toxicity. Additionally, CAR T cells find it difficult to survive and function in immunosuppressive tumor microenvironments^70^. By multiplexing antigens, rupturing TCRs to minimize graft-versus-host disease, or eliminating PD-1, CRISPR allows for the quick optimization of CAR T cells^71^.

By utilizing ‘off-the-shelf’ donor-derived products, allogeneic NK cell treatment seeks to capitalize on their inherent antitumor cytotoxicity^49,72^. Studies evaluating NK cells in AML have shown some encouraging preliminary findings. Activity may be increased by genetic engineering using CARs or inhibitory receptor deletion^73,74^.

### Targeted small molecules

For tumors with such mutations, targeted treatments that inhibit oncogenic drivers such as EGFR, BRAF, and ALK are the standard therapy^75,76^. Nonetheless, resistance frequently appears gradually. Novel drugs, such as osimertinib for EGFR-mutant lung cancer, aid in the fight against resistance mutations^77^. KRASG12C inhibitors at last target KRAS, which has been deemed ‘undruggable’ for a long time and is mutated in around 30% of lung and colorectal malignancies^78^.

### Immunotherapy combinations

Combinations of immunotherapy with chemotherapy, radiation, or targeted medications are becoming more popular than sequential regimens^79^. In melanoma, combined BRAF/MEK inhibition improves anti-PD-1 effectiveness^80^. In lung cancer, adding chemotherapy increases survival with anti-PD-L1^81^. Heterogeneity may be overcome by rational combinations matched to resistance mechanisms.

### Advanced radiation modalities

Tight radiation conformality has been made possible by technological advancements, maximizing tumor dosage while sparing adjacent tissues. Ablative doses are delivered to areas such as the liver and lungs in short fractions of time using stereotactic body radiation treatment (SBRT). Anatomical alterations are accommodated by MR-guided adaptation. Immunotherapy and radiation treatment together may work in concert to stimulate antitumor immunity^74^.

### Targeted alpha therapy

Alpha radiation’s limited range and great potency are utilized in targeted alpha treatment (TAT). In certain advanced solid tumors, the response rates of antibodies tagged with 225Ac and 211At are encouraging^71^. Alpha radiation emitters may have less off-target effects than beta emitters. The best doses and combinations are still being worked out.

### Antibody-drug conjugates

By delivering dose-intensive chemotherapy directly to cancer cells, antibody-drug conjugates (ADCs) minimize systemic exposure^34^. Auristatins, compounds that damage DNA, and pyrrolobenzodiazepines are the payloads of recently authorized ADCs. Target antigens such as CD19, HER2, and BCMA are expressed by lymphoma, breast cancer, and other cancers in which ADCs have action^53^. Overcoming resistance mechanisms and managing toxicity are ongoing issues.

## Exosomal therapies in cancer treatment

**Figure 7:**
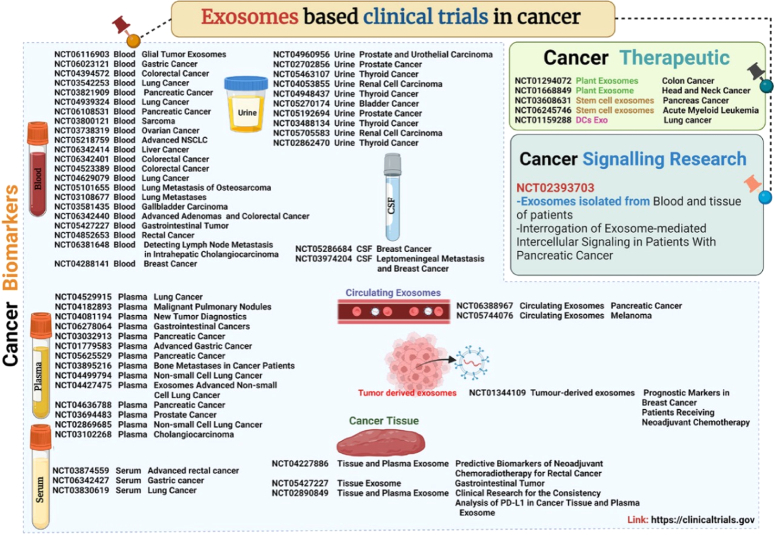
Illustration for exosomes based clinical trials for serum, plasma, blood, and urine samples. (Adapted from Sonar^87^ under CC BY 4.0, Wiley Publishers).

Exosome-based therapies have become a viable area of research for cancer therapy, leveraging the unique properties of extracellular vesicles (EVs) such as exosomes. These nanoscale membrane vesicles, including microvesicles, apoptotic bodies, and exosomes, are generated by various cells, displaying evolutionary conservation^82^. Identified in 1983, exosomes, the smallest vesicle type (on an average 100 nm in diameter), originate from multivesicular endosomes, which get released upon have become a viable area of research for plasma membrane confusion. The innate benefits of using exosomes for therapy make them particularly attractive for clinical applications, especially in cancer diagnosis and treatment^66^. Their small size facilitates tissue penetration, negative zeta potential promotes extended circulation, and immune system resistance enhances their viability^68^. Notably, exosomes play a crucial role in cellular communication and contribute to chemoresistance within the tumor microenvironment. Exosomal drug loading employs two main techniques: passive loading and active loading. Passive loading, involving diffusion, is achieved by incubating drugs with exosomes or exosome-secreting cells, and it is nondestructive^83^. On the other hand, active loading, utilizing methods like sonication and electroporation, enhances drug diffusion efficiency. Studies have demonstrated the effectiveness of active loading, with enhanced cellular drug uptake compared to passive loading methods. Breast carcinoma, known for its heterogeneity and prevalence, has been a focal point for various exosomal therapeutic approaches. Engineered exosomes with doxorubicin and an αv integrin-specific iRGD peptide have shown improved drug penetration, reduced tumor aggression, and decreased tissue toxicity^84^. Similar success has been observed in utilizing exosomes loaded with erastin for triple-negative breast cancer, showcasing enhanced drug solubility and reduced renal toxicity. In pulmonary cancer, exosomal therapies loaded with paclitaxel (PTX) have shown promise, leveraging the overexpression of the sigma receptor in pulmonary metastases^85^. Additionally, the phase I clinical trial investigated the safety and effectiveness of giving patients with advanced non-small-cell lung cancer (NSCLC) dendritic cell-loaded exosomes that contain antigenic peptides presented by HLA^86^ (Fig. 7). Pancreatic cancer, known for its high lethality, has become a subject of exploration for exosomal therapies. Extracellular vesicle (EV) interaction with components of the immune system has been emphasized, and engineered exosomes have demonstrated control of advanced pancreatic ductal adenocarcinoma (PDAC) in mice^88^. Exosomal transportation of the antitumor properties of curcumin between pancreatic cancer cells has also been demonstrated. Prostate cancer, a widespread and frequently deadly male disease, has been targeted for exosomal therapeutics, with paclitaxel enrichment in cancer cell-derived exosomes displaying improved cytotoxicity. Exosomal versus liposomal doxorubicin administration indicated higher cellular accumulation of exosomal doxorubicin near the tumor site^89^. In T cell leukemia, exosomal therapies utilizing aptamers like sgc8 have shown promise, demonstrating reduced cellular cytotoxicity as well as enhanced cellular accumulation^90^. Osteosarcoma, a relatively uncommon bone cancer, has undergone examination for doxorubicin’s effectiveness in fibroblast-derived MG63 cell lines, showing increased cytotoxicity on tumor cells with reduced toxicity to myocardial cells^91^. Liver carcinoma has witnessed significant progress through exosome-biomimetic nanoparticles utilizing porous silicon nanoparticles (PSiNPs) enveloped in biocompatible exosomes. This innovative approach aims to enhance targeted cancer chemotherapy with doxorubicin, resulting in enhanced tumor accumulation, improved penetration, and increased cellular uptake^92^. Skin cancer has been addressed through the utilization of exosomes derived from mesenchymal cells in targeted drug delivery for photodynamic therapy (PDT). These studies demonstrate minimal off-target photosensitizer accumulation, outstanding tumor selectivity, and an increase in overall survival due to PDT^93^. Exosome-based delivery techniques have also been investigated to improve the tumoricidal action of acridine orange (AO)^94^. In conclusion, exosome-based therapies exhibit tremendous potential across a range of cancer types, providing innovative solutions to address the complexities of diagnosis and treatment. These advancements underscore the adaptability and efficacy of exosomal therapies, positioning them as promising tools in the changing field of cancer therapeutics. To harness the potential advantages of nanoparticle-based vaccines, a diverse array of formulations is currently under development. These include virus-like particles, liposomes, polymeric nanoparticles, nanogels, lipid nanoparticles, emulsions, exosomes, and inorganic nanoparticles. Each type of nanoparticle exhibits unique characteristics that researchers are leveraging to create innovative vaccines with applications in both cancer immunotherapy and infectious disease prevention^95^. Vaccines work by introducing a small, inert portion of a particular microbe into the body so that an immune response is evoked^96^.

## CAR T cell therapy

Chimeric antigen receptor T cells, or CAR T cells, represent a groundbreaking advancement in cancer treatment. Engineered with a four-component structure comprising a spacer domain, transmembrane domain, intracellular signaling/activation domain, and single-chain variable fragment (scFv) for target binding^97^. CAR T cells provide a revolutionary approach by recognizing tumor antigens present on the cell surface independently of HLA, which leads to antigen-specific proliferation of T cells, its activation, and cytokine production to combat tumor^98^. This HLA-independent recognition broadens the clinical applications of CAR-T therapy, setting it apart from T cell receptor (TCR) modified cells.

Through the various methods used in gene transfer, it is possible to control T cells to express CARs or chimeric antigen receptors, leading to either transient or permanent modifications in the cells. The integration of vectors derived from lentiviruses or retroviruses in the host genome facilitates stable transduction, while RNA insertion enables transient expression without causing genomic modifications. In ongoing clinical trials, the predominant approach involves the non-PBMC, or peripheral blood mononuclear cells, which are used to amplify specific T cells *in vitro* to produce a significant number of modified T cells^99^. Various in-vitro cell culture systems are employed for amplification, resulting in diverse T cell subsets with varying proportions of memory, naive, and effector T cells. Recognizing the potential significance of T cell subset composition for persistence and replication, wherein a few research teams introduce a step of selection to enrich memory T cells in central circulation^100^. Over more than two decades, researchers have delved into the engineering of T lymphocytes in order to express CARs targeting tumor antigens. Clinical research that took place at the University of Pennsylvania as pioneering research, which showcased notable success in treating refractory advanced chronic lymphocytic leukemia (CLL) with the aid of using anti-CD19 CAR T cells, causing two complete responses in each of the three afflicted people. Four years later, the group’s follow-up studies on the same kind of cancer showed a 57% overall response rate.

Recent investigations underscore the remarkable achievement of using in hematological cancers, particularly acute lymphoblastic leukemia (ALL), CAR T cells have been linked to have resulted in a 90% rate of complete remission and have sustained the effects for up to 2 years. This success has spurred numerous clinical trials which target a group of hematological antigens, such as CD30, CD22, CD20, and CD19^101^. Notably, compared to isolated CD8, unselected T cells, or CD4 T cells alone, CAR T cells, which are made up of CD4 helper T lymphocytes from a pool of naive helper T cells and CD8 cytotoxic T cells from central memory cytotoxic T cells in an equivalent ratio exhibited a higher efficiency in a murine lymphoma model^102^. However, the survival and proliferation of these altered T cells in patients’ peripheral blood is consistently linked to the therapeutic effectiveness of CAR T cells. The difficulties associated with poor in-vivo growth and durability have hampered therapeutic advances following the infusion of modified T cells. Due to the restricted expression of CD19 on the surface of mature B cells rather than other hematopoietic or nonhematopoietic organs, it has emerged as a key target for immunotherapy in B cell lymphomas. In patients who are diagnosed with ALL, CLL, and various B cell lymphomas, the targeting of CD19 by CAR T cells has demonstrated objective regression. Noteworthy advancements have been achieved compared to conventional therapies like chemotherapy or radiotherapy, with CAR T cell trials focusing on the surface protein CD19, which has successfully exhibited lasting positive clinical outcomes. Despite the fact that the majority of early-phase research studies have focused on B cell malignancies, just a handful have targeted solid tumors, and the most effective CARs in B cell malignancies have generally been those which were specific for CD19.

CAR T cell therapy encounters formidable challenges. Initially, obstacles such as the surrounding stroma impede CAR T cell invasion into tumors, where tumorigenic fibroblasts and myeloid cells aid in the development of a fibrous extracellular matrix, impeding T cell penetration^75^. Strategies such as using FAP-CAR T cells have shown improved CAR T cell activity by decreasing tumor fibroblast populations. Furthermore, including the ECM-degrading enzyme heparinase (HPSE) in CAR T cells improves the infiltration of T cells and anticancer efficiency^76^. Following this, the immunosuppressive tumor microenvironment (TME) presents substantial challenges after CAR T cells reach the tumor. The TME, comprising suppressive immune cells, molecular factors, and checkpoint inhibitory proteins, poses hindrances to CAR T cell antitumor immune function^103^. Overcoming immune suppressor cells like Tregs, MDSCs, and TAMs, along with inhibitory proteins like PD-L1, is crucial. Strategies involving the secretion of anti-PD-L1 antibodies by promising outcomes have been observed in the reduction of tumor development and the enhancement of non-T cell antitumor immune subsets’ infiltration by CAR T cells^77^.

Furthermore, the safety and selectivity of the CAR T cells are paramount considerations. Diverse targeting strategies, such as using two different CARs with distinct signaling functions, aim to heighten the specificity of CAR T cell therapy. Overcoming these challenges necessitates innovative genetic modifications and strategic approaches to optimize CAR T cell therapy, ensuring improved efficacy for managing solid tumors. Physical obstacles, immunosuppressive TME elements, and improving CAR T cell specificity are pivotal steps toward advancing the success of this promising therapeutic approach in the realm of cancer treatment^78^.

## Stem cell regenerative therapy

In the realm of human biology, the zygote previously stood out as the singular totipotent stem cell capable of instigating the development of a creature in its entirety through transdifferentiating. In contrast, cells originating from the embryo’s inner cell mass (ICM) exhibited pluripotency, possessing the capability to differentiate into cell types representing the three germ layers while excluding extraembryonic tissues. The state of function of pluripotency factors, including SOX2, cMYC, KLF44, NANOG, and OCT4, determined the stemness and transdifferentiation potential of various stem cells, be they embryonic, extraembryonic, fetal, or adult^79^.

Inducing pluripotency factors through either ectopic expression or functional restoration had the potential to terminally developed cells can be epigenetically modified to become induced pluripotent stem cells (iPSCs). The many types of stem cells include bone marrow stem cells (BMSCs), umbilical cord stem cells (UCSCs), mesenchymal stem cells (MSCs), embryonic stem cells (ESCs), and tissue-specific progenitor stem cells (TSPSCs)

Thomson’s isolation of human ESCs in 1998 marked a pivotal milestone, as these pluripotent cells could give rise to over 200 cell types, promising therapeutic applications across a multitude of diseases. ESC transplantation and transdifferentiation into diverse cell types such as pacemaker cells, hepatocytes, cardiomyocytes, chondrocytes, cones, pancreatic progenitors, and retinal ganglion cells, underscored their potential in regenerative therapeutics. For example, the transdifferentiation of ESCs into retinal cone cells, facilitated by incorporating the COCO gene, held the potential for addressing age-related macular degeneration. ESCs also demonstrated success in cases of spinal cord injuries, enhancing body control and sensation in paraplegic or quadriplegic patients^82^. The spinal cord injury (SCI) pathogenesis, the mechanisms underlying the therapeutic potential of mesenchymal stem cells (MSCs) in SCI, and the potential of stem cell-based therapies as promising avenues for treatment for SCI are reported^104^.

In addressing cardiovascular issues, the regenerative potential of ESC-derived cardiomyocytes surpassed that of several kinds of stem cells. Tissue-specific progenitor stem cells (TSPSCs) played a pivotal role in maintaining tissue homeostasis through controlled cell division. This section delved into the therapeutic applications of various TSPSCs, such as pancreatic progenitor cells, spermatogonial stem cells, skin-derived precursors, adipose-derived stem cells, intestinal progenitor cells, intestinal progenitor cells, limbal progenitor stem cells, and epithelial progenitor stem cells. Instances like the correction of a defective gene in cultured epidermis for a patient with an epidermal blistering disorder showcased the integration of gene therapy with stem cell transplantation^105^. Recognizing the challenges associated with stem cell tourism, the review stressed the imperative need for globally agreed-upon and enforced regulations to safeguard patients.

In 2009, the U.S. Food and Drug Administration authorized the first clinical trial with human participants ESCs, focusing on evaluating the security of spinal cord damage healing using oligodendrocytes produced from ESCs. The existence of numerous human ESC lines, coupled with ongoing efforts in cell banking, provided opportunities for optimal immunological matching. Nevertheless, the potential utilization of the patient’s own cells was used to create induced pluripotent stem cells (iPSCs), offered an alternative that eliminated the necessity for immunosuppression^106^.

**Figure 8:**
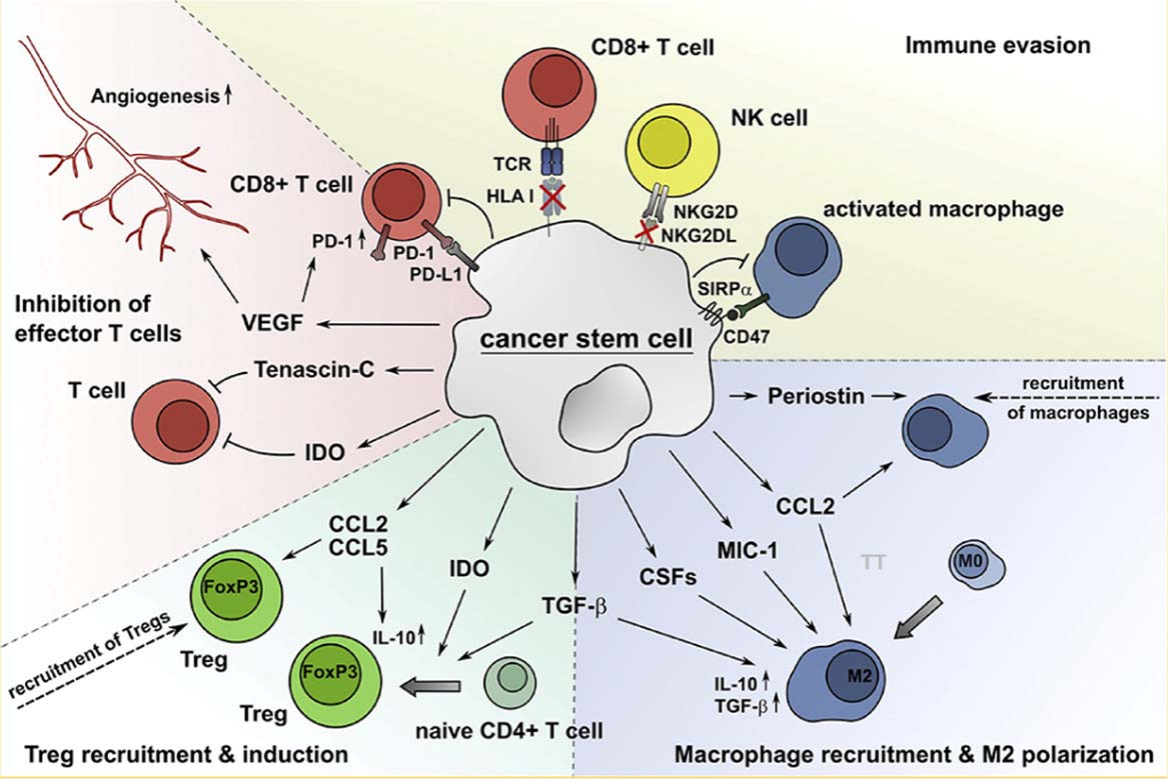
Cancer stem cells (CSCs) manipulate the immune system to evade detection and destruction. They lure and reprogram immune cells like macrophages into allies, suppress T cell activity with various molecules (IDO, TGF-β), and cloak themselves by reducing recognition markers (HLA, NKG2DL). Additionally, they exploit checkpoints (PD-L1/PD-1) to further inhibit T cell function and express CD-47 to avoid being engulfed by macrophages. Through this multi-pronged attack, CSCs create a supportive environment for tumor growth while evading immune elimination. (Adapted from Müller *et al*.^79^ under CC BY, Frontiers Publishers).

Introducing an alternative strategy, it is possible to encourage endogenous stem cells to proliferate or differentiate, mirroring natural processes seen in skin wound healing. The reprogramming of adult mice’s pancreatic exocrine cells develops into useful beta cells that produce insulin, illustrating the potential for tissue repair through cellular reprogramming in situ. Additionally, the discussion expanded to include biomaterials, encompassing resorbable scaffolds and hydrogels, offering clinical avenues for tissue repair by manipulating the stem cell microenvironment. While most clinical applications of stem cells necessitated substantial development time, the immediate applications in drug discovery took center stage^107^. Adult tissue stem cells, ESCs, and iPSCs have been found useful in screening for compounds stimulating self-renewal or specific differentiation programs. The incorporation of stem cell-based assays in drug discovery endeavors not only enhanced the identification of drugs selectively targeting cancer stem cells but also promised more effective and less harmful cancer treatments. The emergence of patient-specific iPSCs as a valuable tool was highlighted for uncovering underlying disease mechanisms. Significant advancements in clinical applications and fundamental research of stem cells in regenerative medicine and other domains have occurred in recent years, motivating individuals to delve deeper into the study of stem cells^108^. In conclusion, stem cells hold significant potential for treating various human diseases and repairing tissue damage, contingent upon addressing concerns of efficacy, safety, and affordability with diligence and advancing our understanding of stem cell biology^109^ (Fig. 8).

## MSCs in autoimmune diseases

Mesenchymal stem cells (MSCs) exhibit unique immunological traits; they only display HLA-I antigens on their surface and lack CD80, CD86, and HLA-II co-stimulatory molecules, which are essential for T cell activation. This immune profile makes them less susceptible to cytotoxic T cell lysis and inefficient in inducing allogeneic lymphocyte proliferation^110^. While there is an agreement that MSCs *in vivo* are mostly immune-inert, ongoing research has revealed complexities, with rodent studies indicating potential immune responses. Despite these nuances, there’s compelling evidence supporting MSCs’ immunomodulatory properties, with the ability to cross allogeneic barriers and dampen immune responses. This has fueled numerous clinical trials exploring MSCs’ potential in treating immune-related diseases and complications arising from transplantation^111^.

Notably, MSCs exert potent immunomodulatory effects on both innate and adaptive immune system components. Studies have demonstrated their impact on T, B, and NK cells, influencing proliferation and functionality. Moreover, MSCs play a pivotal role in shifting the ratio of regulatory T cells (Tregs) to effector/memory T cells, promoting a tolerogenic immune response. Additionally, MSCs modulate antigen-presenting cells, such as dendritic cells (DCs), affecting maturation, differentiation, and cytokine profiles. Mechanistically, with a diverse array of soluble molecules, MSCs’ range of immunomodulatory responses includes transforming growth factor (TGF)-β1, hepatocyte growth factor (HGF), and indoleamine 2,3-dioxygenase (IDO). Despite advancements, the intricacies of MSCs’ interactions with the immune system warrant further exploration, especially considering their therapeutic potential in autoimmune disorders and transplantation settings^84^

Crucially, MSCs harness three molecules from fetomaternal tolerance IDO, HLA-G, and LIF to exert their immunomodulatory effects. IDO, a key enzyme mediating immunosuppression, plays a central role in Treg generation. HLA-G, known for its role in fetal-maternal tolerance, influences immune cell function, with surface expression of HLA-G1 contributing to T cell inhibition by MSCs. LIF, initially associated with pregnancy tolerance, also proves vital in MSC immunomodulation, inhibiting effector T cells and promoting Treg generation^11^. Additionally, factors like tissue origin and environmental conditions influence MSC behavior, requiring careful consideration in therapeutic applications. Furthermore, the activation of Toll-like receptors (TLRs) on MSCs by pathogen-associated molecular patterns (PAMPs) or inflammatory cues contributes to their immunomodulatory functions, adding another layer of complexity to their interactions with the immune system. Understanding these nuances is crucial for optimizing MSC-based immunotherapies and advancing their clinical applications^112^.

Stem cell therapy has evolved into a crucial life-saving intervention for a spectrum of life-threatening diseases, with transplanting bone marrow historically been used to treat hematological diseases and more recently extended to nonmalignant diseases such as hematopoietic anomalies and congenital immunodeficiencies, metabolic errors, and autoimmune diseases. The application of hematopoietic stem cell therapy (HSCT) is reserved for severe cases due to the inherent complications associated with this treatment modality, illnesses like congenital immunodeficiencies and hematological abnormalities, and susceptibility to infections. Notably, post-transplant infections, particularly bacterial and viral, stand out as significant impediments to patient survival following stem cell transplantation^113^.

In an endeavor to enhance the effectiveness of stem cell therapy, researchers are directing efforts toward minimizing GVHD while simultaneously preserving immunologic integrity and mitigating infection risks. A key focus involves manipulating stem cell grafts, employing strategies such as the enrichment of hematopoietic stem cells (HSCs), depletion of T lymphocytes implicated in GVHD, and the purging of cells affected by pathology. The spotlight is also on mesenchymal stem cells (MSCs) due to their favorable characteristics, including ease of culture, expansion, and potential for gene therapy. Within the bone marrow microenvironment, stromal cells play a pivotal role in facilitating durable engraftment of stem cell grafts and aiding in the process of immune reconstitution, effectively addressing major challenges in the realm of stem cell therapy^114^.

Treating autoimmune illnesses, which impact over 3% of the U.S. population, presents complex difficulties. Stem cell treatment, especially when it comes to autologous bone marrow transplants, emerges as a promising approach for addressing conditions like systemic lupus erythematosus (SLE) and other autoimmune disorders. These diseases are characterized by inflammatory processes, autoreactive T cells, and defective autoantibodies^115^. The essence of stem cell therapy lies in its capacity to replace malfunctioning cells, providing a steady stream of medicinal substances and acting as a target for gene therapy. Autologous stem cell transplantation using bone marrow or peripheral blood stem cells presents distinctive advantages such as rapid availability and reduced dependence on immune suppressive conditioning^116^.

Delving specifically into the context of systemic lupus, studies conducted in autoimmune-prone mouse models illuminate the intricate function of stem cell treatment. Transplanting purified HSCs or bone marrow that has been devoid of T cells from healthy donors emerges as an effective strategy for preventing and treating autoimmune diseases^117^.

The contribution of stromal cells within the graft to engraftment and recovery from autoimmunity underscores the critical importance of comprehending the roles played by both HSCs and MSCs in the intricate landscape of stem cell therapy for autoimmune diseases. Addressing defects in HSCs, particularly in the context of systemic lupus, stands out as a promising avenue based on experimental models, offering valuable insights into the potential applications of stem cell therapy for a spectrum of autoimmune conditions^118^.

## Stem cell therapy in neurodegenerative diseases

Stem cell therapy has transformed the medical landscape in recent decades, demonstrating its efficacy in treating various organ-related diseases. The clinical applications of stem cells have steadily expanded, providing hope for addressing neurodegenerative disorders such as amyotrophic lateral sclerosis (ALS), Alzheimer’s, Parkinson’s disease (PD), and Huntington’s disease (HD). Stem cell therapy, particularly in PD, capitalizes on the capacity of stem cells to develop into neurons that produce dopamine, a pivotal mechanism in mitigating the gradual decrease of the number, shape, or function of neurons. Notably, research has shown successful outcomes in both preclinical and clinical trials involving the transplantation of human embryonic mesencephalic tissue in PD patients^119^.

Recent advances in genetic engineering have facilitated the differentiation of dopaminergic neurons from various stem cell sources, including rat neural stem cells (NSCs), mouse fibroblasts, and human embryonic stem cells (ESCs)^120^. Noteworthy findings, such as the reprogramming of fibroblasts from PD patients into dopaminergic neurons, underscore the potential of stem cell therapy in addressing PD’s clinical challenges. While some double-blinded trials yielded negative results, emphasizing the importance of precise stem cell collection, factors influencing dopaminergic neuron development have been identified. These factors, such as OTX2, Nurr1, tyrosine hydroxylase, FGF8, and others, are essential in controlling stem cell differentiation in Parkinson’s disease^97^.

The application of stem cells in Huntington’s disease (HD) has emerged more recently, with experiments exploring neural regeneration and behavioral benefits in phenotypic HD models. MSCs genetically engineered to overexpress BDNF or nerve growth factor demonstrated reduced behavioral deficits in mouse models of HD. Furthermore, mouse NSCs acting as growth factor (GDNF) delivery vehicles in murine HD models showcased promising results in reducing neuronal death and associated motor impairments. Human multipotent stromal cells from bone marrow implanted into the hippocampus of HD mice exhibited enhanced proliferation and neural differentiation of endogenous NSCs^121^.

Research in HD monkeys has identified dental pulp stem cells as potential sources for personal stem cells, offering advantages in terms of multipotent differentiation and reduced immunosuppressive therapy post-transplant. While the exploration of stem cell therapy in HD is less extensive compared to PD, these findings highlight its potential in ameliorating neurodegenerative processes and enhancing neural regeneration^122^.

The disease known as amyotrophic lateral sclerosis (ALS), which causes both lower and upper motor neuron loss, presents unique challenges for stem cell therapy due to its unknown pathogenesis and disease progression mechanisms. The primary goal in ALS stem cell therapy is the substitution of motor neurons, achieved through the regulation of inflammation and expression of neurotrophic factors. Clinical trials, including a phase I trial with neural stem cells (NSCs) implanted into the spinal cord of ALS patients, have focused on safety assessments^123^. Stem cell transplantation into the frontal motor cortex and dorsal spinal cord has shown promising safety outcomes, although the determination of optimal cellular types and anatomical sites for implantation remains an ongoing challenge.

With dementia, Alzheimer’s disease (AD) is the most common kind and poses challenges due to late-stage diagnosis and the involvement of oxidative stress and inflammation in its pathogenesis. Stem cell therapy, studied in mouse models of AD, demonstrated memory improvement and cognitive function via BDNF-mediated responses^124^. When NSCs were transplanted into the hippocampal regions of elderly Down syndrome mice, tau/reelin-positive granules significantly decreased. This suggests that alterations in granule density might be used to evaluate the effectiveness of treatment interventions. The ongoing endeavor to produce induced pluripotent stem cells (iPSCs) unique to each patient and linked to AD suggests a growing investigation into stem cell treatment for this intricate neurodegenerative illness^125^.

**Figure 9:**
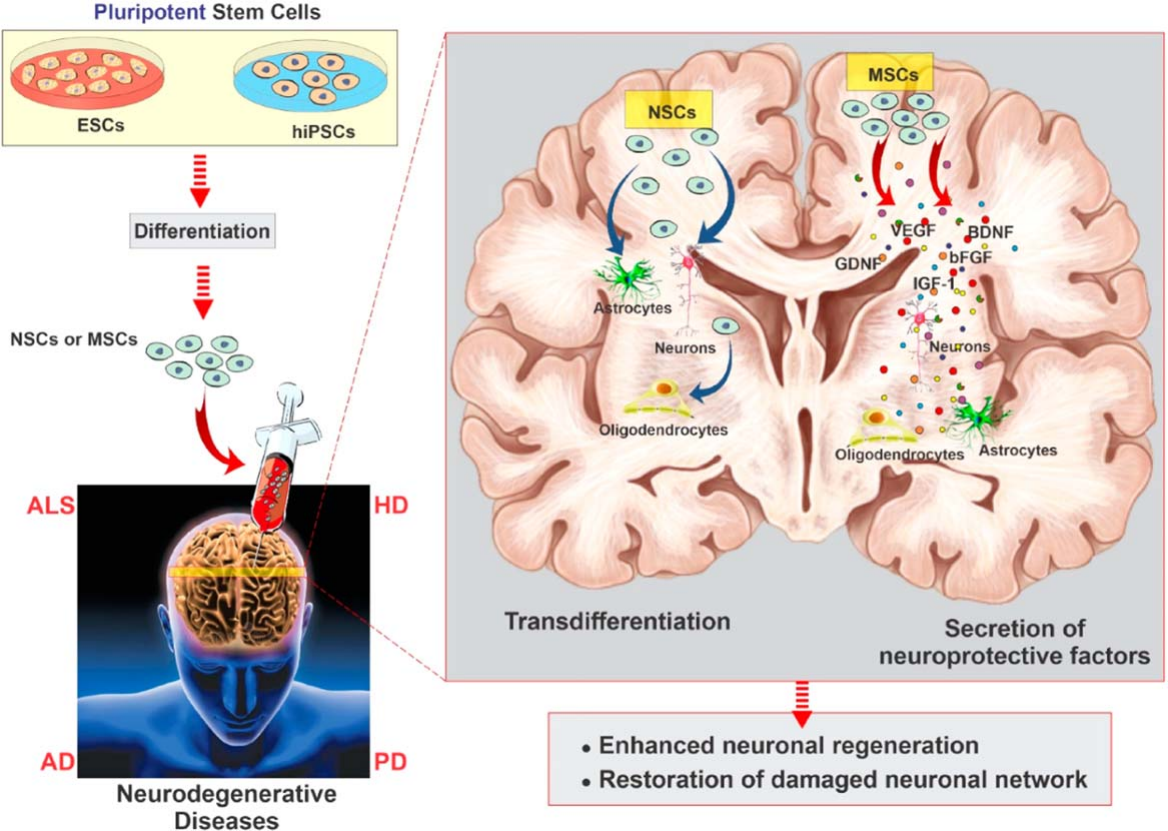
Neurodegenerative disease modeling of hiPSCs and ESCs. The action of MSCs, including the secretion of growth and neurotrophic factors, can act as a coadjutant to nervous tissue regeneration by promising angiogenesis, neurogenesis, and immunomodulation. (Adapted from Sivandzade and Cucullo^129^ under CC BY 4.0 from MDPI Publishers).

Many clinical studies are investigating different aspects of stem cell therapies for neurodegenerative disorders^126,127^. The data so far appear to support the results obtained from preclinical studies to some extent. For instance, there is a consensus of data showing that the secretion of growth factors (such as brain-derived neurotrophic factor, glial cell line-derived neurotrophic factor, and nerve growth factor) achieves neuroprotection. This fundamental mechanism is responsible for the observed improvements in neurodegenerative disorders^128^. Additionally, there is significant evidence showing that stem cell therapies can enhance neurogenesis in neurodegenerative patients (Fig. 9).

## The use of nanotechnology in stem cell-based therapy

Induced pluripotent stem cells (iPSCs) provide a customized method for self-therapies. Stem cells are the source of donor cells in regenerative medicine. Numerous studies have demonstrated the efficacy of neural stem cell (NSC)-mediated therapy for neurological illnesses, suggesting that it may be used to address a range of neuron malfunctioning concerns. Despite the therapeutic advantages identified in NSCs, their clinical application faces significant hurdles, notably in terms of screening NSC migration to damaged tissues, real-time imaging-guided treatment *in viv*o, and directed differentiation. These difficulties result from the intricate relationships between NSCs and several internal and external elements, including the extracellular matrix (ECM), neighboring cells, growth hormones, and inflammation at sites of injury^120^. Resolving these difficulties is critical to enabling the larger-scale clinical deployment of NSC treatment. The unique characteristics of nanomaterials, including their high surface-to-volume ratio, surface energy, and unique mechanical, thermal, electrical, magnetic, and optical behaviors, make them attractive instruments for breaking through obstacles in the field of brain stem cell treatment. Adding nanomaterials directly to the culture medium, coating culture containers, and conjugating nanomaterials with certain scaffolds for 3D culture systems are some of the ways that nanomaterials may be used to improve the effectiveness of stem cell culture systems. Once nanoparticles (NPs) are internalized, their contact with the membrane of stem cells or other intracellular components alters cellular signaling pathways^130^.

In stem cell research, nanotechnology plays a crucial role, particularly in the development of substrates for large-scale stem cell production. The emphasis has been on amplifying neural cells, which holds substantial implications for advancing therapies to treat neurodegenerative diseases. Achieving procedures involving cell transplantation needs bulk cultivation of cells that effectively maintain their undifferentiated state during manufacturing. The distinct physicochemical properties of metallic nanoparticles (NPs), such as the presence of high-energy atoms on their surface, make them very promising. Numerous research works demonstrate the significant impact that metallic nanoparticles (NPs) have on the growth and development of various cell types, including stem cells. A variant of iron oxide NPs known as superparamagnetic iron oxide (SPIO) NPs has characteristics similar to superparamagnetic, making them a prospective tool for the treatment of regenerative diseases^131^. By upregulating cell cycle-related proteins, SPIO-NPs like Ferucarbotran can enhance cell cycle progression and encourage the growth of human mesenchymal stem cells (hMSCs). Graphene (G) and graphene oxide (GO) have become popular substrates for the cultivation of induced pluripotent stem cells (iPSCs) because of their ultra-large surface area, 2D structure, and great biocompatibility^132^.

**Figure 10:**
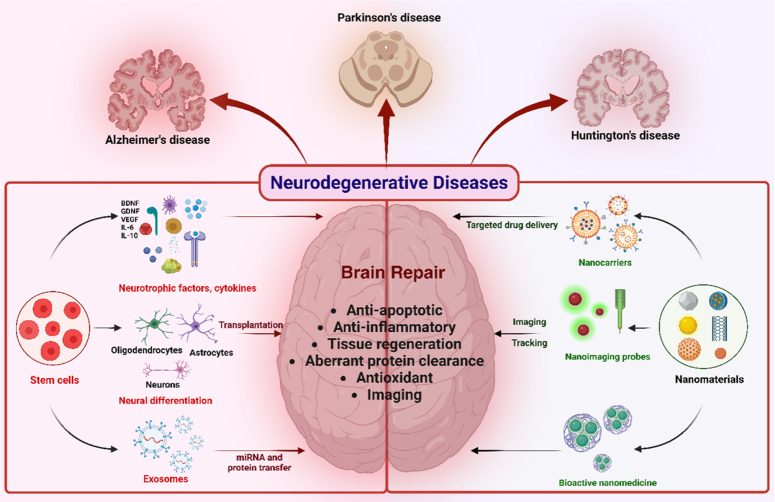
Schematic illustration of the cooperation of nanomaterials and stem cell therapies for brain repair in neurodegenerative diseases. (Adapted from Wei *et al*.^133^ under CC BY NC from Dove Press Publisher).

Applying nanoparticles (NPs) and nanoengineered substances is known as nanomedicine, offers a strategic approach to modulate neural stem cell differentiation. Biomaterials, including nanoparticles, present advantages in regenerative medicine over existing media by facilitating cell growth and differentiation into specific lineages and providing a platform for the production of patient-specific tissues (Fig. 10). Tissue-specific stem cells, when combined with nanoparticle-including biomaterials, enhance differentiation into specific mature cells or tissues while maintaining undifferentiated states and self-renewal activities. Biodegradable and self-assembling nanoparticles, including poly(β-amino esters), are highly effective in transfecting human embryonic stem cells (hESCs) with plasmid DNA^134^. Combining tissue engineering technology with stem cell-based strategies holds potential for producing regenerative medicine, contributing to the replacement of damaged or wounded tissues for therapeutic purposes. In response to certain stimuli, tissue-specific stem cells can dedifferentiate, redifferentiate, or transdifferentiate, emphasizing the role of nanotechnology in the regenerative process^134^.

For successful therapeutic tissue replacement, enhancing cellular-tissue interactions through a cellular biochemical cue and favorable physical surroundings must work together. With their iron oxide core and magnetic coating, superparamagnetic iron oxide nanoparticles (SPIONs) have certain qualities that help achieve this objective^135^. Conjoining the non-covalent interaction of functional proteins and nanostructures is facilitated by nanomaterial compounds containing SPIONs co-covered with photonic ZnO^136^. This improves stem cell differentiation and the catalytic activity of important proteins in the stem cell cycle. Various complexes of NP-RA, involving polymeric nanoparticles loaded with retinoic acid, emerge as potent neurogenic agents for vascular diseases of neurodegenerative disorders in conjunction with vasculature^137^. These advances highlight the potential of stem cell therapy combined with nanotechnology in the advancement of regenerative medicine and controlled neurogenesis.

## Conclusion

The emerging field of stem cell therapy stands at the forefront of transforming cancer treatment, autoimmune diseases, and advancing neurobiology. The ability of stem cells to differentiate into specialized cell types offers unprecedented potential in regenerative medicines, particularly in repairing damaged tissues and organs ravaged by cancer or neurological disorders. By harnessing the regenerative and transdifferentiation capacity of stem cells, researchers are pioneering novel therapeutic strategies for personalized cancer therapies, mitigating the side effects of conventional therapies and potentially in the treatment of cancer. Moreover, in neurobiology, stem cell research is unraveling the complexities of the nervous system, offering insights into neurobiological disorders. The convergence of CRISPR therapy, stem cell advancements, and nanotechnology represents a paradigm shift in cancer treatment and neurobiology research. These innovative approaches hold immense promise for addressing longstanding challenges in medicine. Furthermore, nanotechnology has enabled precise drug delivery and imaging capabilities at the molecular level. Nanoparticles loaded with drugs can target cancer cells specifically, minimizing systemic toxicity and enhancing treatment efficacy. In neurobiology, nanotechnology facilitates the study of neuronal function and the development of novel therapies that can cross the blood-brain barrier to effective treatment. Together, these innovations underscore a transformative era in biomedicine where interdisciplinary collaboration between geneticists, oncologists, neuroscientists, and engineers is driving breakthroughs at an unprecedented pace. In conclusion, while the potential of these technologies is yet to be realized, their combined impact on cancer treatment and neurobiology is promising. Continued research and clinical trials will be crucial in translating these advancements from bench to bedside, ultimately improving outcomes for patients and expanding our understanding of human health and disease.

## Ethical approval

Not applicable.

## Consent

Not applicable.

## Source of funding

The authors extend their appreciation to the Deanship of Scientific Research at King Khalid University for funding this work through a large group Research Project under grant number RGP2/406/45.

## Author contribution

P.M. and N.M.: conceptualization; D.B., S.M., and A.P. and A.B.: writing original draft and editing; S.W.K., A.S.,T.A., and H.A.S.: data collection, analysis, and interpretation; N.M. and P.M.: supervision.

## Conflicts of interest disclosure

The authors declare no conflicts of interest.

## Research registration unique identifying number (UIN)

Not applicable.

## Guarantor

Nobendu Mukherjee.

## Data availability statement

No new data are generated.

## Provenance and peer review

Invited Special Paper for the special issue ‘Evolving trends in stem cell therapy’.
